# Supplementary material for: An open-source framework for large-scale, flexible evaluation of biomedical text mining systems
Source: J Biomed Discov Collab. 2008 Jan 29;3:1. doi: 10.1186/1747-5333-3-1 (PMC2276192; doi:10.1186/1747-5333-3-1)
Supplement: Additional file 1 — Evaluation platform source code. The additional file contains the software evaluation framework discussed in this paper. The most current release can be downloaded from SourceForge.net [1]. See the accompanying README file for instructions on installation and use. Also included in the distribution is a collection of UIMA wrappers for some commonly used BioNLP tools and annotated corpora. [file 1747-5333-3-1-S1.zip › bionlp_uima_repository-1.0.1/docs/api/edu/uchsc/ccp/util/nlp/parser/corpus/IProLinkParser.html]

IProLinkParser (Center for Computational Pharmacology Util/UIMA API)


|  |  |  |
| --- | --- | --- |
| **Overview** \| **Package** \| **Class** \| **Tree** \| **Deprecated** \| **Index** \| **Help** | |  |
| **PREV CLASS**   **NEXT CLASS** | **FRAMES**    **NO FRAMES**     **All Classes** |  |
| SUMMARY: NESTED \| FIELD \| CONSTR \| METHOD | DETAIL: FIELD \| CONSTR \| METHOD |  |


---


## edu.uchsc.ccp.util.nlp.parser.corpus Class IProLinkParser

```
java.lang.Object
  edu.uchsc.ccp.util.nlp.parser.corpus.IProLinkParser
```

---

``` public class IProLinkParser extends Object ```

This class contains methods to parse the 2 IProLink XML corpora files. Specifically, this class is usefull if you want to process the corpus all at
once, and then retrieve all of the annotations. Use the other classes in this package to process the documents one at a time.

**Author:**
:   williamb

---

**Constructor Summary**

`IProLinkParser()`


| **Method Summary** | |
| --- | --- |
| `static List<String[]>` | `getDocuments()` |
| `static List<TextAnnotation>` | `getTextAnnotations()` |
| `static void` | `main(String[] args)`             args[0] = iProLink corpus1 file  args[1] = iProLink corpus2 file |
| `static void` | `parseIProLinkCorpus1(BufferedReader br)`             corpus1.xml has tags: protein, acronym, and compound-protein |
| `static void` | `parseIProLinkCorpus2(BufferedReader br)`             corpus2.xml has tags: protein and long-form |

**Methods inherited from class java.lang.Object**

`clone, equals, finalize, getClass, hashCode, notify, notifyAll, toString, wait, wait, wait`

**Constructor Detail**

### IProLinkParser

```
public IProLinkParser()
```


**Method Detail**

### parseIProLinkCorpus1

```
public static void parseIProLinkCorpus1(BufferedReader br)
                                 throws IOException
```

:   corpus1.xml has tags: protein, acronym, and compound-protein

    :   **Parameters:**: `iProLinkCorpus1File` -: `connectionPropertiesFile` - **Throws:**: `IOException`

---


### parseIProLinkCorpus2

```
public static void parseIProLinkCorpus2(BufferedReader br)
                                 throws IOException
```

:   corpus2.xml has tags: protein and long-form

    :   **Parameters:**: `iProLinkCorpus2File` -: `connectionPropertiesFile` - **Throws:**: `IOException`

---


### getTextAnnotations

```
public static List<TextAnnotation> getTextAnnotations()
```

---


### getDocuments

```
public static List<String[]> getDocuments()
```

---


### main

```
public static final void main(String[] args)
```

:   args[0] = iProLink corpus1 file  
    args[1] = iProLink corpus2 file  

    :   **Parameters:**: `args` -


---


|  |  |  |
| --- | --- | --- |
| **Overview** \| **Package** \| **Class** \| **Tree** \| **Deprecated** \| **Index** \| **Help** | |  |
| **PREV CLASS**   **NEXT CLASS** | **FRAMES**    **NO FRAMES**     **All Classes** |  |
| SUMMARY: NESTED \| FIELD \| CONSTR \| METHOD | DETAIL: FIELD \| CONSTR \| METHOD |  |


---
